# Causes and consequences of fine-scale population structure in a critically endangered freshwater seal

**DOI:** 10.1186/1472-6785-14-22

**Published:** 2014-07-09

**Authors:** Mia Valtonen, Jukka U Palo, Jouni Aspi, Minna Ruokonen, Mervi Kunnasranta, Tommi Nyman

**Affiliations:** 1 Department of Biology, University of Eastern Finland, Joensuu, Finland; 2 Laboratory of Forensic Biology, Hjelt Institute, University of Helsinki, Helsinki, Finland; 3 Department of Biology, University of Oulu, Oulu, Finland; 4 Institute for Systematic Botany, University of Zurich, Zurich, Switzerland

**Keywords:** Cryptic population structure, Effective population size, Gene flow, Genetic erosion, Landscape genetics, Small population

## Abstract

**Background:**

Small, genetically uniform populations may face an elevated risk of extinction due to reduced environmental adaptability and individual fitness. Fragmentation can intensify these genetic adversities and, therefore, dispersal and gene flow among subpopulations within an isolated population is often essential for maintaining its viability. Using microsatellite and mtDNA data, we examined genetic diversity, spatial differentiation, interregional gene flow, and effective population sizes in the critically endangered Saimaa ringed seal (*Phoca hispida saimensis*), which is endemic to the large but highly fragmented Lake Saimaa in southeastern Finland.

**Results:**

Microsatellite diversity within the subspecies (*H*_E_ = 0.36) ranks among the lowest thus far recorded within the order Pinnipedia, with signs of ongoing loss of individual heterozygosity, reflecting very low effective subpopulation sizes. Bayesian assignment analyses of the microsatellite data revealed clear genetic differentiation among the main breeding areas, but interregional structuring was substantially weaker in biparentally inherited microsatellites (*F*_ST_ = 0.107) than in maternally inherited mtDNA (*F*_ST_ = 0.444), indicating a sevenfold difference in the gene flow mediated by males *versus* females.

**Conclusions:**

Genetic structuring in the population appears to arise from the joint effects of multiple factors, including small effective subpopulation sizes, a fragmented lacustrine habitat, and behavioural dispersal limitation. The fine-scale differentiation found in the landlocked Saimaa ringed seal is especially surprising when contrasted with marine ringed seals, which often exhibit near-panmixia among subpopulations separated by hundreds or even thousands of kilometres. Our results demonstrate that population structures of endangered animals cannot be predicted based on data on even closely related species or subspecies.

## Background

Efficient management of endangered animal populations requires information on the species’ biology and behaviour, but also on census size, effective size, population structure, and migration rates. Especially in the absence of incoming migration and gene flow, fragmentation of an already small population may increase its extinction risk by exacerbating demographic and genetic stochasticity in the even smaller subpopulations [1]. Gene flow may be prevented by physical barriers [2] or by a lack of suitable corridors connecting habitat patches [3], but sometimes fine-scaled spatial substructuring may result from purely behavioural patterns, *e.g*., site fidelity [4,5], social systems [6,7], or individual-level foraging specialisation [8].

Studying spatial connectivity and levels of gene flow in rare, elusive, and endangered mammals is difficult using traditional approaches like mark–recapture or telemetry methods [9]. To substitute and complement traditional methods, a number of genetic tools have been developed. While keeping in mind that genetic methods typically provide little information on the demographic consequences of interpopulation migration [10], they are nevertheless a powerful and often the only tool for assessing connectivity within and among populations [11].

The landlocked Saimaa ringed seal (*Phoca hispida saimensis*) provides an excellent model system for studying how small population size and spatial subdivision influence key genetic and demographic parameters and processes. This endemic ringed seal subspecies derives from marine seals that became trapped in Lake Saimaa in southern Finland after the last glacial period, *i.e*., nearly 10,000 years ago [12]. The Saimaa ringed seal population experienced a serious anthropogenic bottleneck during the 20^th^ century [13,14] and, despite a post-1980s recovery, the population still numbers only *c*. 300 seals [15]. Previous studies [16,17] have shown that the Saimaa ringed seal is genetically very depauperate in terms of both nuclear and mitochondrial variability. As the population has remained completely isolated for hundreds of generations, the trajectory of its gene pool is determined largely by population size and internal population structure.

**Figure 1:**
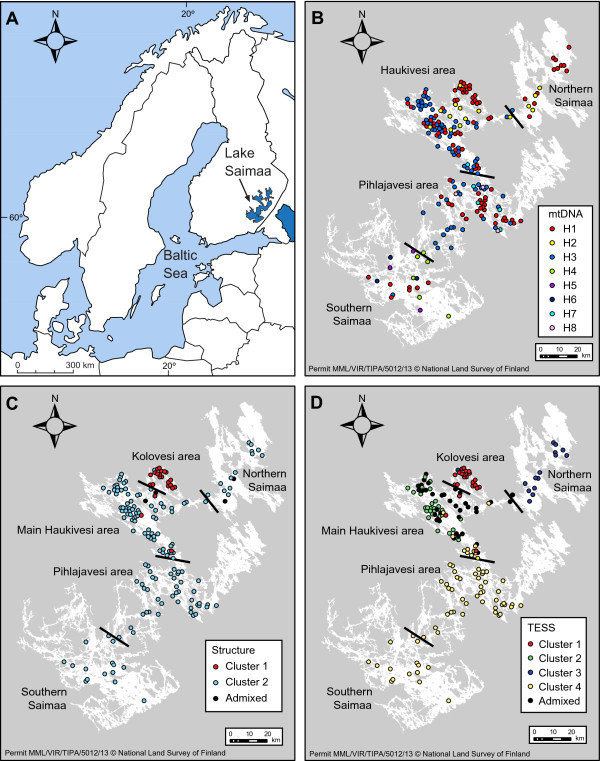
**Location of Lake Saimaa in Finland (A) and collection sites of the ringed seal specimens (B – D).** Initial **(B)** and updated **(C, D)** regional division used in this study. Different colours imply different mtDNA haplotypes **(B)**, and different clusters identified by Structure **(C)** and TESS **(D)** (see Figure 5A,C).

Lake Saimaa extends nearly 200 km in the north–south direction and covers over 4,400 km^2^[18], but the lake is very fragmented, with narrow straits separating its main basins (Figure 1). Telemetry studies have shown that adult Saimaa ringed seals are relatively sedentary [19,20], but have also indicated that population connectivity could be maintained by dispersal of immature individuals [21]. A scenario of substantial gene flow was supported by Palo *et al*. [16], showing weak differentiation at microsatellite loci between the northern and southern parts of the lake. However, Valtonen *et al*. [17] recently reported strong mtDNA differentiation among the four main basins of Lake Saimaa. These contradictory results could be explained by the low amount of data in the earlier microsatellite assessment, but also by sex-biased gene flow.

Here we have examined the genetic diversity, population structure, and dispersal patterns in the Saimaa ringed seal based on data from 172 individuals genotyped at 17 microsatellite loci. We first tested the hypothesis that subpopulations in the four main breeding areas within the lake are differentiated. As the initial analysis revealed autosomal differentiation between the main regions, we proceeded to investigate the spatial distribution of genetic variation in more detail, taking advantage of the specific collection locations of the genotyped individuals. Using also previously published mtDNA data [17], we then explored isolation-by-distance patterns, migration rates among subpopulations, presence of sex-biased gene flow, local effective population sizes, and temporal trends in diversity. Especially when contrasted with recent analogous studies on marine ringed seals, our results provide insights into factors that induce population subdivision, and into the effects of spatial structuring on the genetic composition of small, isolated populations.

## Methods

### Laboratory analyses

We used tissue specimens from Saimaa ringed seals found dead and deposited in a tissue bank maintained by the University of Eastern Finland and Natural Heritage Services of Metsähallitus [22]. The majority of the sampled individuals were pups (<1 yrs; 76%), and the main cause of death of both adults and pups was entanglement in fishing gear (66%). All specimens had been stored at -20°C.

Total genomic DNA was extracted using DNeasy Blood and Tissue Kits (Qiagen) following the manufacturer’s instructions. Microsatellite loci were amplified using primers originally designed for grey seal (*Halichoerus grypus*) [23-26], leopard seal (*Hydrurga leptonyx*) [27], and harbour seal (*Phoca vitulina*) [28,29] (Additional file 1: Table S1). PCR reactions were set up in 10 μl volumes, each containing 25 – 50 ng genomic DNA, 1 μM each primer, 0.5 U AmpliTaq Gold DNA polymerase (Applied Biosystems), 1X PCR buffer, 1.25 – 1.5 mM MgCl_2_, and 0.1 mM of each dNTP (Finnzymes). The thermal cycler profile consisted of initial denaturation at 94°C for 3 min, followed by 30 – 35 cycles of denaturation at 94°C for 30 s, annealing at 50 – 58°C for 30s, and extension at 72°C for 30s, with a final extension step at 72°C for 5 min (Additional file 1: Table S1). PCR products were run on an ABI 3730 DNA Analyzer (Perkin Elmer Applied Biosystems), and allele peaks scored using GeneMapper v. 4.0 (Applied Biosystems). Micro-Checker v. 2.2.3 [30] was used to test for the presence of null alleles, allelic dropout, and scoring errors due to stuttering, with Bonferroni-adjusted 95% confidence intervals. The frequency of null alleles at each locus was estimated using the software FreeNA [31].

The microsatellite dataset (Additional file 2: Table S2) was supplemented by, and contrasted with, mtDNA sequence variation in 215 individuals from the same population, obtained by Valtonen *et al*. [17]. The 704-bp mtDNA fragment spans the 5′ domain of the mitochondrial control region.

### Spatial and temporal division of specimens

The genotyped individuals originate from different parts of Lake Saimaa and represent a time span of three decades (1980 – 2008). In order to analyse the spatial genetic structure of the population, the specimens were initially divided into four regional samples following the topography of the lake (Figure 1B): Northern Saimaa (microsatellites: *N*_ms_ = 15, and mtDNA: *N*_mt_ = 19), Haukivesi area (*N*_ms_ = 99 and *N*_mt_ = 116), Pihlajavesi area (*N*_ms_ = 43 and *N*_mt_ = 61), and Southern Saimaa (*N*_ms_ = 15 and *N*_mt_ = 19).

In addition, the individuals were divided into three temporal samples based on their collection decade: the 1980s (*N*_ms_ = 59 and *N*_mt_ = 79), 1990s (*N*_ms_ = 48 and *N*_mt_ = 54), and 2000s (*N*_ms_ = 65 and *N*_mt_ = 82). A decade was considered an appropriate time span to detect temporal changes, since the estimated generation time of the ringed seal is *c*. 11 years (estimated by Palo *et al*. [16] after Smith [32]).

Genetic diversity indices were also calculated across an alternative temporal division scheme, in which individuals were grouped based on their year of birth: 1965–1979 (*N*_ms_ = 14), the 1980s (*N*_ms_ = 54), the 1990s (*N*_ms_ = 47), and the 2000s (*N*_ms_ = 54) (see [17] for details on age determination).

### Estimation of genetic diversity and effective population size

We used Arlequin v. 3.5.1.2 [33] to estimate the number of alleles (*A*), observed (*H*_O_) and expected (*H*_E_) heterozygosities, and Wright’s inbreeding coefficients (*F*_IS_) for each spatial and temporal sample. Departures from Hardy–Weinberg equilibrium and the presence of linkage disequilibrium between pairs of loci were tested with Arlequin and GENEPOP v. 4.1.3 [34]. Estimates of allelic richness (*A*_R_) were obtained using the rarefaction method implemented in HP-Rare [35]. *A*_R_ was estimated separately for each spatial and temporal sample, based on the smallest sample size within each division scheme. Individual observed heterozygosities (*H*_O_) were calculated using IR macroN4 ( [36]; http://www.zoo.cam.ac.uk/directory/william-amos), and the relationship between heterozygosity and year of birth was tested by performing a linear regression analysis in SPSS Statistics v. 19 (IBM).

Effective sizes for the total population, as well as for different regional and temporal subsamples within the lake, were estimated using two different methods. First, we used the single-sample method based on linkage disequilibrium implemented in the software LDNe [37], while assuming a lowest allele frequency of 4% in order to prune singletons also from the smallest regional samples (= Northern and Southern Saimaa). Second, utilising the sampling from different decades, we estimated *N*_e_ with the temporal method in TempoFS [38], assuming Waples sampling scheme 1 (see [39] and references therein). For both methods, 95% confidence intervals were obtained through jackknifing. The sample size from the Haukivesi area allowed also estimating effective population sizes separately for different decades.

### Assessments of population structure

Genetic differentiation among the spatial and temporal samples was investigated using analysis of molecular variance (AMOVA) [40,41] and by estimating pairwise *F*_ST_ values between samples using Arlequin. For the two regions with the largest sample sizes (Haukivesi and Pihlajavesi), we also ran a hierarchical AMOVA with spatial samples subdivided into temporal subsamples, and *vice versa*. Significance levels were estimated on the basis of 10,000 permutations. Because *F*_ST_ is influenced by the level of heterozygosity (*H*_S_) in the studied subpopulations [42-44], we used GenAlEx v. 6.501 [45,46] to estimate overall spatial differentiation also based on *D*_est_[42] and *G*"_ST_[44], which correct for genetic diversity as well as the number of subpopulations in the analysis; departures from zero were inferred based on 999 permuted datasets. To facilitate comparisons between the microsatellite and mtDNA datasets (see [47]), we estimated global *D*_est_ also for the mtDNA sequence data using a script [48] employing the seqinr [49] and ape [50] packages in R 3.0.1 [51]; statistical significance was assessed based on 10,000 permutations of the data. The distribution of microsatellite variation across individual seals from the main regions of the lake was visualized with factorial correspondence analysis (FCA) implemented in the program GENETIX v. 4.05.2 [52]. The statistical significance of differences in FCA axis 1 and 2 scores among regional groups were tested using a multi-response permutation procedure (MRPP) test in PC-ORD v. 5.33 [53].

Genetic structuring of the population was further studied using Bayesian genotype-assignment approaches. Analyses in Structure v. 2.3.4 [54,55] were run without prior information on the sampling locality. We employed the admixture model with correlated allele frequencies, and implemented a burn-in period of 100,000 followed by 500,000 MCMC iterations. We set the number of clusters (*K*) from 1 to 8, and ran 20 replicate analyses per each *K* to ensure convergence. Subsequently, we determined the most likely number of clusters by estimating the logarithm of the data probability given each number of *K* (log*P*(*X*|*K*); [54]), and on the basis of Δ*K* values estimated using the *ad hoc* approach of Evanno *et al*. [56] implemented in Structure Harvester v. 0.6.93 [57].

Because collection locations were known for all but one of the sampled 172 seals, we used TESS v. 2.3.1 [58,59] for a more detailed evaluation of spatial differentiation within the lake. In these analyses, individual sampling-site coordinates were included as prior information. Before the analyses, we removed some links from the default neighbourhood system created by the program, in order to improve its match to the geography of Lake Saimaa (Additional file 3: Figure S1). Thereafter, we ran TESS using a no-admixture model with 200,000 iterations, of which the first 100,000 were excluded as a burn-in, to test the number of clusters (*K*) from 2 to 8, with ten replicates for each *K*. Plots of the deviance information criterion (DIC) against *K* were used to identify the most likely number of clusters, which was then used in 100 replicate runs with the admixture model, using the same number of iterations as above. Finally, we averaged results from the ten runs having the highest likelihoods using CLUMPP v. 1.1.2 [60], and visualised the results using Distruct v. 1.1 [61]. The initial hypothesis concerning the regional division of the Saimaa ringed seal population, which was based on the topography of the lake, was then reassessed according to the Structure and TESS results.

The presence of an isolation-by-distance (IBD) pattern among seals within the lake was tested by using SPAGeDi v. 1.3 [62] to contrast the degree of relatedness among individuals with their geographic distances on a logarithmic scale [63]. Using the aforementioned location information, we estimated IBD for the microsatellite (*N* = 171) and mtDNA (*N* = 214) datasets using the kinship coefficient of Loiselle *et al*. [64], which is considered suitable for datasets containing rare alleles [65]. For mtDNA, we also used *N*_ij_, a kinship analogue based on genetic distances among haplotypes [66]. Between-haplotype distances were estimated based on a TrN + I + Γ substitution model using parameter values from Valtonen *et al*. [17]. In all three analyses, ten spatial distance classes were created using the equal-frequency method, which generates uneven distance intervals with roughly equal numbers of pairwise comparisons. Significance levels for the mean kinship coefficient (departure from zero) for each distance class, as well as for the regression slope (*b*), were obtained by 10,000 permutations, and standard errors were estimated by jackknifing over loci.

### Estimation of migration rates and sex-biased patterns of gene flow

Migration rates among Lake Saimaa regions were estimated using the Bayesian multilocus genotyping method implemented in BayesAss v. 1.3 [67]. We performed 1 × 10^6^ burn-in iterations followed by 9 × 10^6^ iterations with a sampling frequency of 2,000, with Δ parameters of allele frequency, migration rate, and inbreeding coefficient set to 0.15, 0.17, and 0.17, respectively, to achieve the recommended acceptance rates of between 40% and 60%. The analysis was repeated three times with different initial seed numbers to ensure convergence.

In order to infer whether dispersal within the lake is sex-biased, we estimated the relative amount of male and female gene flow following the approach of González-Suárez *et al*. [68], which takes into account the different mode of inheritance of mitochondrial and nuclear genes, as well as their differing numbers of gene copies of within a population (see also [69]). This indirect method was chosen because the small number of subadults and adults in the data [22] prevented direct assessments of sex-specific differentiation. We first estimated the amount of male differentiation using equation 1b in González-Suárez *et al*. [68], and then assessed the ratio of male and female gene flow rates based on equation 2c in the same paper.

## Results

### Data characterization, genetic diversity, and effective population size

Genotypes for all 17 microsatellite loci were obtained for 168 individuals, while four individuals lacked data for one locus each (Additional file 2: Table S2) [22]. Significant heterozygote deficit was observed at eight loci, but the finding was not consistent through regional or temporal samples. Over all samples, Micro-Checker analyses suggested presence of null alleles at six loci (*Hg1.4*, *Hg3.6*, *Hg6.1*, *Hg8.9*, *Hgdii*, and *Pvc78*) with low frequencies (*r* < 0.1) in all cases, most likely due to population substructure (see below). Scoring errors suggested for the loci *Hg3.6* and *Hg8.9* were ruled out by independent manual rechecking of the data by two researchers. In Arlequin, consistent and significant linkage disequilibrium in all temporal samples after a sequential Bonferroni correction was found between loci *Hg4.2* and *Pvc78,* whereas Bonferroni-corrected GENEPOP results indicated no disequilibrium. We consider the latter result more reliable, because the permutational likelihood-ratio test implemented by Arlequin assumes that all loci are in Hardy–Weinberg equilibrium [70], which is not the case here (Additional file 2: Table S2). Therefore, the main analyses were performed using the full 17-locus dataset.

The average number of alleles per locus was 3.5, but the range was broad, so that 16 loci had 2 to 4 alleles, while 16 were found in locus *Hl15* (Table 1 and Additional file 2: Table S2). The low allelic diversity was reflected in the very low overall level of expected heterozygosity in the population (*H*_E_ = 0.36 ± 0.22).

**Table 1:** Measures of genetic diversity in spatial and temporal samples of the Saimaa ringed seal population based upon analyses of 17 microsatellite loci

**Sample**	**N**	***N***_**P**_	***N***_**A**_ **± SD**	***A***_***R***_	***H***_**O**_ **± SD**	***H***_**E**_ **± SD**	***F***_**IS**_
Lake Saimaa	172	17	3.47 ± 3.32	–	0.33 ± 0.21	0.36 ± 0.22	0.075***
Northern Saimaa	15	14	2.29 ± 1.16	2.27	0.34 ± 0.27	0.33 ± 0.24	-0.004
Haukivesi area	99	15	3.24 ± 3.15	2.70	0.35 ± 0.23	0.38 ± 0.23	0.074***
*Kolovesi*	*20*	*13*	*2.41* ± *1.66*	*2.32*	*0.37* ± *0.31*	*0.36* ± *0.28*	*-0.031*
*Main Haukivesi area*	*79*	*15*	*3.18* ± *2.92*	*2.63*	*0.34* ± *0.23*	*0.35* ± *0.24*	*0.024*
Pihlajavesi area	43	15	2.59 ± 1.42	2.26	0.31 ± 0.25	0.30 ± 0.23	-0.034
Southern Saimaa	15	14	2.47 ± 1.42	2.45	0.31 ± 0.24	0.30 ± 0.22	-0.045
yod 1980s	59	17	3.29 ± 2.62	3.21	0.35 ± 0.23	0.37 ± 0.23	0.054*
yod 1990s	48	17	3.29 ± 2.85	3.29	0.34 ± 0.22	0.37 ± 0.23	0.077*
yod 2000s	65	17	3.06 ± 2.38	2.96	0.32 ± 0.20	0.35 ± 0.22	0.092***
yob 1965-1979	14	16	2.94 ± 1.75	2.94	0.38 ± 0.25	0.38 ± 0.23	-0.019
yob 1980s	54	17	3.06 ± 2.38	2.71	0.34 ± 0.23	0.37 ± 0.23	0.075**
yob 1990s	47	17	3.29 ± 2.85	2.69	0.33 ± 0.22	0.36 ± 0.23	0.073*
yob 2000s	54	17	3.06 ± 2.38	2.58	0.32 ± 0.19	0.35 ± 0.22	0.100**

*N*_P_, number of polymorphic loci; *N*_A_, average number of alleles per locus; *A*_R_, allelic richness; *H*_O_, observed heterozygosity; *H*_E_, expected heterozygosity; *F*_IS_, inbreeding coefficient; yod, year of death; yob, year of birth. Statistically significant *F*_IS_ values are marked with asterisks: ***P < 0.001; **P < 0.01; *P < 0.05.

Regional estimates are shown both for the initial four-region division, and for the updated five-region scheme in which Haukivesi is split into the Kolovesi and Main Haukivesi areas (in italics).

**Figure 2:**
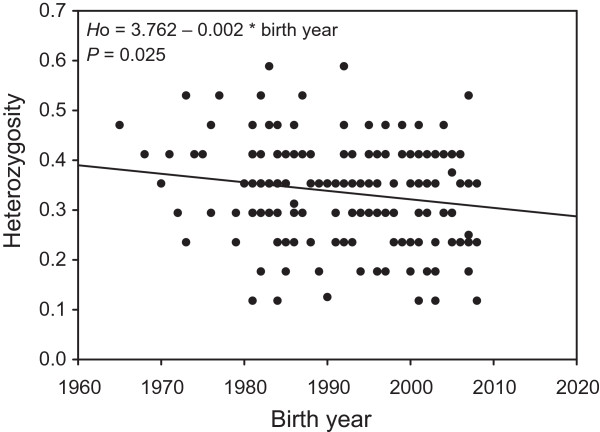
Observed heterozygosity of Saimaa ringed seal individuals in relation to their year of birth.

No obvious differences in genetic diversity were found among regions (Table 1). The data also suggested diversity decrease along the decades, but there is not enough statistical power for testing the pattern. However, there was a weak but statistically significant negative relationship between individual observed heterozygosity and year of birth (*H*_O_ = 3.762–0.002*birth year; *r*^2^ = 0.030, *P* = 0.025; Figure 2). Temporal *F*_IS_ estimates increased over the study period from effectively zero for seals born before 1980 to a significantly positive *F*_IS_ = 0.100 (*P* = 0.001) in the 2000s (Table 1).

Estimates of effective population sizes, based on both linkage disequilibrium and temporal changes of allele frequencies, were extremely low (Table 2). For the total population, the LD-based estimate was *N*_e_ = 14.7 (95% CI 9.9 – 20.7), and similarly low numbers were obtained for different decades, ranging from *N*_e_ = 10.7 (6.1 – 17.4) in the 1990s to *N*_e_ = 32.7 (18.6 – 66.1) in the 2000s. Estimates based on the temporal approach were somewhat higher, but with broad confidence intervals: 1980s – 1990s *N*_e_ = 69 (33 – inf.); 1990s – 2000s *N*_e_ = 53 (31 – 171); and *N*_e_ = 113 (51 – inf.) over two generations (1980s – 2000s). Effective population sizes within the four main regions ranged from 4.7 to 45.9 (Table 2). The highest estimate (yet with a rather broad confidence interval) was found in the Pihlajavesi area and the lowest in Northern Saimaa. As for the whole population, *N*_e_s within Haukivesi were highest during the 2000s.

**Table 2:** Effective population sizes for the total Saimaa ringed seal population as well as for regional and temporal samples based on linkage disequilibrium and temporal changes in allele frequencies

	**Linkage disequilibrium**				**Temporal method**		
	**All**	**1980s**	**1990s**	**2000s**	**1980s – 1990s**	**1990s – 2000s**	**1980s – 2000s**
Lake	14.7	12.3	10.7	32.7	69	53	113
Saimaa	(9.9 – 20.7)	(8.1 – 18.1)	(6.1 – 17.4)	(18.6 – 66.1)	(33 – inf.)	(31 – 171)	(51 – inf.)
Northern	4.7	–	–	–			
Saimaa	(2.1 – 13.6)						
Haukivesi	8.8	3.3	4.5	30.7	61	24	92
area	(5.6 – 12.3)	(2.5 – 5.3)	(2.7 – 7.6)	(15.7 – 86.1)	(17 – inf.)	(12 – inf.)	(47 – 1873)
* Kolovesi*	*10.7*	–	–	–			
	*(4.6 – 27.0)*						
* Main*	*21.8*	*10.8*	*7.5*	*53.6*	*46*	*15*	*59*
* Haukivesi area*	*(12.1 – 40.3)*	*(6.2 – 19.5)*	*(3.0 – 15.4)*	*(22.5 – 2366.1)*	*(15 – inf.)*	*(8 – 135)*	*(23 – inf.)*
Pihlajavesi	45.9	–	–	–			
area	(20.6 – 251.3)						
Southern	15.3	–	–	–			
Saimaa	(6.0 – 100.1)						

inf. = infinity. Estimates are shown both for the initial four-region division, and for the updated five-region scheme in which Haukivesi is split into the Kolovesi and Main Haukivesi areas (in italics). 95% confidence intervals are given in parentheses.

### Population structure in space and time

**Figure 3:**
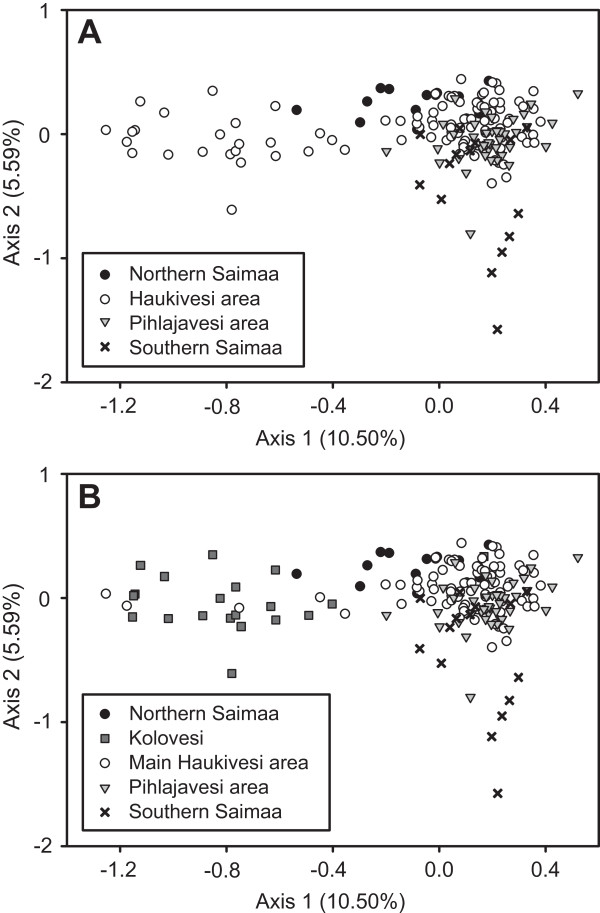
**Factorial correspondence analysis (FCA) plot of ringed seals from different regions of Lake Saimaa.** Individuals are marked with different symbols based on **(A)** the initial division to four main regions and **(B)** on the updated division to five regions (see legends).

**Figure 4:**
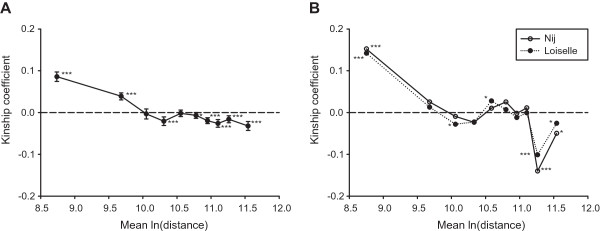
**Average kinship coefficient plotted against logarithmic distance between Saimaa ringed seal individual pairs.** The plots are based on **(A)** 17 microsatellite loci, and **(B)** mtDNA haplotypes (Loiselle) and genetic distance between haplotypes (*N*_ij_). Distance classes differing significantly from the mean kinship of the population are marked with asterisks: ***P < 0.001; *P < 0.05.

In microsatellite analyses, significant overall differentiation was detected among the four main regions of Lake Saimaa (*F*_ST_ = 0.065, *D*_est_ = 0.043, *G*"_ST_ = 0.121; all *P* ≤ 0.001), and the result was confirmed by pairwise *F*_ST_ estimates among regions (Table 3). These patterns can be seen in the FCA plot, in which seals from the same region tended to cluster together, even though there was wide overlap among groups (Figure 3A). FCA axis loads of the regional groups differed significantly from each other (MRPP test, *P* < 0.001), and all pairwise comparisons were statistically significant (*P* < 0.01 in each case). Microsatellite differentiation was moderate in comparison to estimates based on mitochondrial control-region sequences, which were consistently higher for overall differentiation (*F*_ST_ = 0.390, *P* < 0.001 [17]; *D*_est_ = 0.256, *P* = 0.047) as well as for pairwise *F*_ST_ values (Table 3). A highly significant negative relationship between relatedness and spatial distance between pairs of individuals was found using both microsatellites (*b* = -0.039, *P* < 0.001; Figure 4A) and mtDNA (*b*_Loiselle_ = -0.055; *b*_Nij_ = -0.067; both *P* < 0.001; Figure 4B).

**Table 3:** **Genetic differentiation (pairwise *F***_**ST**_**) between regional samples of the Saimaa ringed seal population based on microsatellite and mtDNA variation**

			**Northern**	**Haukivesi**		***Main Haukivesi***	**Pihlajavesi**	**Southern**
	***N***_**ms**_	***N***_**mt**_	**Saimaa**	**area**	***Kolovesi***	***area***	**area**	**Saimaa**
Northern Saimaa	15	19	–	0.454	*0.698*	*0.467*	0.446	0.392
Haukivesi area	99	116	0.039**	–	*–*	*–*	0.389	0.333
*Kolovesi*	*20*	*21*	*0.161*	*–*	*–*	*0.544*	*0.534*	*0.527*
*Main Haukivesi area*	*79*	*95*	*0.047*	*–*	*0.170*	*–*	*0.398*	*0.344*
Pihlajavesi area	43	61	0.096	0.063	*0.236*	*0.057*	–	0.311
Southern Saimaa	15	19	0.153	0.071	*0.209*	*0.075*	0.054	–

*N*_ms_ = sample size in microsatellite analyses; *N*_mt_ = sample size in mtDNA analyses.

Estimates based on microsatellites are given below diagonal and mtDNA variation above diagonal. Results are shown for the initial four-region division, and for the updated five-region scheme in which Haukivesi is split into the Kolovesi and Main Haukivesi areas (in italics). Differentiation in all comparisons was statistically significant at *P* < 0.001 after a sequential Bonferroni correction, except for **P < 0.01.

Overall microsatellite differentiation among temporal samples based on decades (1980s, 1990s and 2000s) was very weak (*F*_ST_ = 0.002, *P* < 0.001), and this was true also for all pairwise comparisons (all *F*_ST_ ≤ 0.002, *P* ≥ 0.293). Hierarchical AMOVA of the two regions with the largest sample sizes (= Haukivesi and Pihlajavesi) likewise showed that, regardless of the order of the hierarchy, most of the differentiation could be attributed to variation among regions and individuals, while the effect of decades was weak and statistically non-significant (results not shown). In contrast, mtDNA-based analyses showed highly significant differentiation among decades (*F*_ST_ = 0.384, *P* < 0.001; see discussion in [17]).

**Figure 5:**
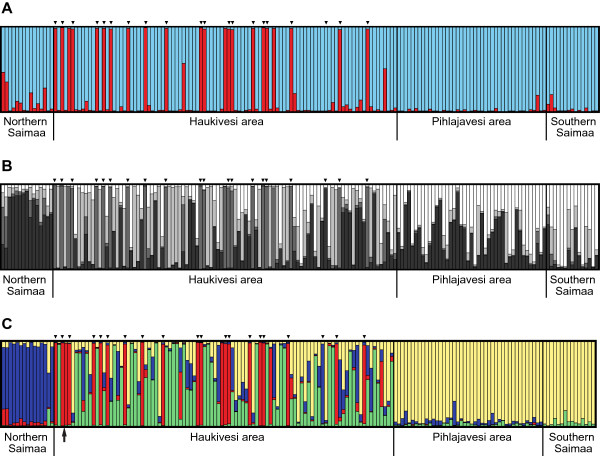
**Assignment of individual Saimaa ringed seals into population clusters based on microsatellite data.** Results are shown as indicated by Structure **(A)** for *K* = 2 and **(B)***K* = 4, and **(C)** by TESS for *K* = 4. Each bar represents a single individual, and the height of each bar represents the relative probability of it belonging to a given cluster. Individuals are grouped by the four main sampling areas, inverted triangles above the plots denote individuals that originate from the Kolovesi part of the Haukivesi area. Cluster colours in **(A)** and **(C)** correspond to the colours used in Figure 1C,D. The arrow below the plot in **(C)** indicates an individual that was excluded from the analysis in TESS due to lacking detailed location information.

In Bayesian assignment analyses in Structure, mean log-likelihood was maximized at *K* = 4 (Additional file 4: Figure S2A), but the Evanno *et al.*[56] approach favoured a two-cluster model (Additional file 4: Figure S2B). In the two-cluster results, 22 individuals were assigned to cluster 1 and 146 to cluster 2 (red and light blue in Figure 5A, respectively), while the assignments of four individuals remained ambiguous (membership coefficient *Q* < 0.7, following, *e.g*., Kopatz *et al.*[71]). Notably, all individuals assigned to cluster 1 originated from the Haukivesi area (Figure 5A) and, upon closer examination, 86% of them were found to be from the Kolovesi basin in northeastern Haukivesi (Figure 1C). The Kolovesi individuals were similarly identifiable in the four-cluster plot, but the geographic pattern of the other three clusters was less apparent (Figure 5B).

In the TESS assignment analyses, DIC reached a plateau at *K* = 4 (Additional file 4: Figure S2C). However, the spatial distribution of individuals in these clusters did not entirely follow our predefined structure (Figures 1D and 5C). Instead, Northern Saimaa was the only predetermined region that was more or less identifiable in its original form in the TESS chart (Cluster 3, blue), while the distribution of Cluster 4 (yellow) individuals roughly corresponds to Pihlajavesi and Southern Saimaa. Cluster 1 (red) individuals were largely restricted to the aforementioned Kolovesi basin (Figure 1D), whereas the rest of the Haukivesi area seemed to represent an admixture zone: although 35% of individuals belonged to Cluster 2 (green), 44% remained weakly assigned.

Based on these results, we reassessed our initial, topography-based division of Lake Saimaa by splitting the Haukivesi area (Figure 1B) into two subregions, Kolovesi and Main Haukivesi (Figure 1C,D). For the rest of the lake, we retained the original division scheme, on grounds of the significantly non-zero pairwise *F*_ST_ values (Table 3). As expected, using the five-region division elevated global estimates of differentiation, based on both microsatellites (*F*_ST_ = 0.107 *vs*. 0.065; *D*_est_ = 0.074 *vs*. 0.043; *G*"_ST_ = 0.196 *vs*. 0.121) and mtDNA (*F*_ST_ = 0.444 *vs*. 0.390; *D*_est_ = 0.298, *P* < 0.001, *vs*. 0.256). Pairwise *F*_ST_ estimates showed that Kolovesi is highly differentiated from the rest of the lake (Table 3), which can also be seen in the five-region FCA plot (Figure 3B; MRPP test, overall *P* < 0.001; pairwise contrasts, all *P* < 0.001). Furthermore, the significantly positive *F*_IS_ = 0.074, *P* = 0.0002 of the undivided Haukivesi area disappeared when Main Haukivesi and Kolovesi were analyzed separately (*F*_IS_ = 0.024, *P* = 0.183; and *F*_IS_ = -0.031, *P* = 0.741, respectively) (Table 1). Separating the Kolovesi and Haukivesi individuals elevated LD-based *N*_e_ estimates in the new Main Haukivesi area, while estimates based on the temporal method decreased (Table 2).

### Migration rates and sex-biased patterns of gene flow

BayesAss runs implementing the initial four-region scheme did not converge, so only results based on the five-region division are reported here. Migration rates among different parts of Lake Saimaa were generally low (Table 4): point estimates of self-recruitment exceeded 87% in four regions, and Southern Saimaa was the only region estimated to receive immigrants at a considerable rate (27.8%). These immigrants seem to originate from the adjacent Pihlajavesi area, since the 20.4% rate of migration from Pihlajavesi to Southern Saimaa was the only interregional estimate that was significantly higher than zero.

**Table 4:** Migration rates of Saimaa ringed seals among five regions of the lake based on 17-locus microsatellite genotypes

**From**				**To**		
	***N***	**Northern Saimaa**	**Kolovesi**	**Main Haukivesi area**	**Pihlajavesi area**	**Southern Saimaa**
Northern Saimaa	15	*0.874 ± 0.114*	0.011 ± 0.013	0.016 ± 0.019	0.004 ± 0.006	0.013 ± 0.016
Kolovesi	20	0.010 ± 0.015	*0.969 ± 0.021*	0.025 ± 0.011	0.002 ± 0.004	0.012 ± 0.015
Main Haukivesi area	79	0.022 ± 0.033	0.006 ± 0.008	*0.888 ± 0.068*	0.003 ± 0.006	0.050 ± 0.052
Pihlajavesi area	43	0.084 ± 0.102	0.009 ± 0.012	0.068 ± 0.067	*0.987 ± 0.012*	**0.204 ± 0.061**
Southern Saimaa	15	0.009 ± 0.014	0.006 ± 0.009	0.003 ± 0.004	0.003 ± 0.005	*0.722 ± 0.033*

The estimates (*m* ± SD) are averaged over three BayesAss runs. Rates are estimated in both directions, from source regions given in the first column ("From") to destination regions in the following columns ("To"). Self-recruitment within each region is shown along the diagonal (in italics), and interregional rates in which 95% confidence intervals do not include zero are in bold.

The expected level of differentiation for paternally inherited genes (*F*_ST(males)_ = 0.099) among the five Lake Saimaa regions was only slightly lower than overall microsatellite differentiation (*F*_ST_ = 0.107), and the same was true for nearly all regional pairs (Tables 3 and 5). The overall ratio of male to female gene flow was 7.26, and ratios estimated for separate regional pairs ranged from 3.87 to 18.55 (Table 5).

**Table 5:** Estimates of sex-specific differentiation and gene flow in the Saimaa ringed seal population

	**Northern**	**Kolovesi**	**Main Haukivesi**	**Pihlajavesi**	**Southern**
	**Saimaa**		**area**	**area**	**Saimaa**
Northern Saimaa	–	15.67	18.55	8.18	3.87
Kolovesi	0.129	–	7.22	5.01	5.44
Main Haukivesi area	0.045	0.142	–	11.31	6.50
Pihlajavesi area	0.090	0.186	0.055	–	7.79
Southern Saimaa	0.143	0.170	0.074	0.055	–

Estimates show the expected level of differentiation for paternally inherited genes (*F*_ST(males)_; below diagonal) and the ratio of male- and female-mediated gene flow (*m*_(males)_/*m*_(females)_; above diagonal) among ringed seals from five regions of Lake Saimaa.

## Discussion

### Genetic diversity and effective population size

The Saimaa ringed seal has very low genetic diversity (*H*_E_ = 0.36) in comparison to marine ringed seal populations, in which microsatellite-based estimates of expected heterozygosities range from *H*_E_ = 0.80 to *H*_E_ = 0.89 [72-74]. Indeed, microsatellite variation within the subspecies is, to our knowledge, the lowest that has thus far been found within the order Pinnipedia in studies in which monomorphic loci have been excluded (*cf*. Mediterranean monk seal (*Monachus monachus*), *H*_E_ = 0.40 [24]; Hawaiian monk seal (*Monachus schauinslandi*), *H*_E_ = 0.49 [75]; spotted seal (*Phoca largha*), *H*_E_ = 0.51 [76]; northern elephant seal (*Mirounga angustirostris*), *H*_E_ = 0.40 [77]), and heterozygosity is low even when contrasted with the mean for placental mammals that have experienced a recent demographic threat (*H*_E_ = 0.50 ± 0.03; [78], see also [79]). The reduced diversity of the Saimaa population is not plausibly explained by ascertainment bias [78,80], which could stem from the use of loci developed for other seal species, since it should similarly affect the levels of diversity in marine ringed seals. On the contrary, heterozygosity estimates in marine ringed seals [72-74] are in most cases higher than in the species for which the loci were originally designed [25,27,29]. Coalescent simulations indicate that the main loss of genetic diversity in the Saimaa ringed seal predates the 20^th^-century anthropogenic bottleneck [16,17], but the question remains as to whether the current population – given its size and spatial structure – will be able to maintain the remaining variability.

Unfortunately, it seems that the answer to this question is "No": Estimates of total and regional effective population sizes were very low, ranging roughly between 5 and 113 (Table 2). Comparable values have been obtained for other isolated and/or bottlenecked populations of large mammals, *e.g.*, Finnish wolves (*Canis lupus*), *N*_e_ = 37.8 – 43.0 [81], Western Canadian mountain goats (*Oreamnos americanus*), *N*_e_ = 23.2 – 65.9 [82], and Iberian lynxes (*Lynx pardinus*), *N*_e_ = 8.5 – 23.1 [79]. It should be noted that historical, long-term *N*_e_s of several hundred, estimated by Palo *et al.*[16] and Valtonen *et al.*[17], are far higher than our contemporary estimates, suggesting that the population has been substantially larger in the past. Notably, estimates based on linkage disequilibrium were clearly lower than those based on temporal changes in allele frequencies (Table 2). This is probably due to population structure lowering LD-based estimates (see [83]), as is indicated by the rise in Main Haukivesi *N*_e_ after exclusion of Kolovesi individuals (Table 2). We therefore consider the higher estimates based on the temporal method to be more applicable for the Saimaa ringed seal. When reflected to the current census size (310 in the year 2012 [15]), the temporal *N*_e_ estimates yield a ratio of *N*_e_/*N*_c_ = 0.17 – 0.36. The lower end of this range is consistent with the average ratio observed in wild populations (0.16), and the higher end would comply with the higher-than-average *N*_e_/*N*_c_ ratios observed in small threatened populations [84].

Nevertheless, even the higher temporal *N*_e_s apparently are not enough for safeguarding the existing diversity: our detailed analysis based on the birth years of the sampled individuals detected slowly declining individual heterozygosity in microsatellite loci during the past few decades (Figure 2), which is in line with the post-1960s decrease in mtDNA variation observed by Valtonen *et al.*[17]. The fact that the overall inbreeding coefficient has risen at the same time (Table 1) suggests that the increasing autozygosity is due to a progressive increase in differentiation among and/or within regions rather than to loss of alleles. Examples of similar trends are accumulating from other species that have undergone recent anthropogenic collapses [85] and range fragmentations [79,86].

### Population structure and gene flow

Gene flow among populations is often restricted or even prevented by geographic barriers, but assessing what constitutes an obstacle for a given species is not always straightforward [11,87,88]. In analyses of spatial genetic variation, a clear benefit of the Saimaa ringed seal population is that the topography of the lake unambiguously determines possible routes of dispersal among subpopulations, and the level of differentiation then depends on the migration rates through these routes. Telemetry studies have shown that adult home ranges may span sites nearly 40 km apart [19] and that even first-year pups can travel up to 15 km per day after being weaned [21]. However, it has hitherto remained unclear whether such occasional long-distance movements lead to gene flow among the main breeding areas.

Our results demonstrate that ringed seal subpopulations inhabiting the main regions of Lake Saimaa are differentiated with respect to both autosomal and mitochondrial variation (see also [17]). However, Bayesian assignment analyses showed that genetic structuring within the lake is more subtle than we originally envisioned, by uncovering semi-isolation of the Kolovesi basin, which essentially constitutes a labyrinthine cul-de-sac separated from the main parts of Haukivesi by a narrow strait (Figure 1C,D). All in all, the level of genetic differentiation among different parts of Lake Saimaa (*F*_ST_ = 0.065 – 0.107) is remarkable considering the short distances among the main basins (Figure 1). As a comparison, Palo *et al*. [72] showed that ringed seals of the Baltic Sea are essentially panmictic (*F*_ST_ = 0.000), and geographic differentiation is weak also in grey seals, in which Graves *et al.*[89] found Saimaa-like *F*_ST_ values only between Baltic and North Sea breeding colonies (*F*_ST_ = 0.068 – 0.097), while differentiation within the Baltic was clearly lower (*F*_ST_ ≤ 0.023). In general, interpopulation genetic differentiation in marine seals is often negligible – or at least lower than that observed within Lake Saimaa – even across thousands of kilometres [72-74,90-92]. A potential caveat here is that the dependence of *F*_ST_ on overall heterozygosity [42,44] means that direct comparisons are complicated by the very disparate levels of genetic diversity in the Saimaa ringed seal and its marine counterparts. We therefore used the approach of Heller & Siegismund [43] and Meirmans & Hedrick [44] to convert overall differentiation indices among marine ringed seal populations estimated by Davis *et al*. [73] (*F*_ST_ = 0.005) and Martinez-Bakker *et al*. [74] (*F*_ST_ = 0.0086) into *G*"_ST_ values, based on the reported *F*_ST_ s, mean values of *H*_S_, and numbers of sampled populations. The converted estimates (*G*"_ST_ = 0.047, and *G*"_ST_ = 0.056, respectively) resulting from these studies, both of which include populations on different continents, are still below the overall differentiation among regions within Lake Saimaa (*G*"_ST_ = 0.121 – 0.196).

As can be expected based on the high spatial differentiation, interregional migration estimates (Table 4) were very low in comparison to values estimated for other large mammals inhabiting fragmented landscapes (*e.g*., [2,11]). Across comparable geographic distances, equally low rates have been detected only for the endangered Ethiopian wolf (*Canis simensis*), in which remaining populations are restricted to mountaintops separated by unsuitable lowlands [3]. Although the migration estimates were generally near zero, it appears that Pihlajavesi, the most important breeding area producing some 40 – 50% of the pups born annually [15], serves as a source for the small Southern Saimaa subpopulation. Assessing whether the estimated migration rates are high enough to maintain demographic connectivity is difficult (*cf*. [10]), but our results suggest at least partial demographic independence of subpopulations, which could significantly increase the risk of stochastic extinction by lowering the demographic effective population size (*N*_d_) well below the census size [93].

Microsatellites exhibited lower levels of interregional differentiation (measured as *F*_ST_ and *D*_est_) and a smoother isolation-by-distance pattern than did mtDNA (Figure 4A,B), suggesting that gene flow is mainly mediated by males, as is often the case in pinnipeds [5,94,95] and mammals in general [96]. The roughly sevenfold male-to-female gene flow ratio is comparable to values reported for, *e.g*., harbour seals [97] and California sea lions (*Zalophus californianus*) [68].

## Conclusions

The critically endangered ringed seal subspecies endemic to Lake Saimaa is genetically impoverished and fragmented into several partially isolated subpopulations. Microsatellite diversity within the subspecies is the lowest recorded so far within the order Pinnipedia and, worryingly, our analyses reveal an ongoing downward trajectory in both microsatellite (this study) and mtDNA variation [17]. Although further studies are needed for investigating whether the low diversity in these presumably neutral markers correctly reflects the level of variation in adaptively important loci, and although genetically based adversities have thus far not been demonstrated in the population, maintaining the existing variability is recommendable, especially considering the need of the subspecies to adapt to a substantially warmer climate in the coming decades (*cf*. [98,99]). The observed diversity declines are fortunately relatively slow, as is expected for a long-lived species with overlapping generations (*cf*. [100-102]). Hence, both negative trends most likely can be reversed if the still-fragile recovery of the population can be sustained and strengthened by conservation actions. Managed translocations of individual seals could restore demographic connectivity (*cf*. [91,103,104]), and would simultaneously aid in protecting the remaining genetic variation; according to our results, such efforts should be concentrated on females, which otherwise seem to be particularly reluctant dispersers.

The level of genetic differentiation among animal populations is a function of multiple factors, including time, effective population sizes, degree of geographic separation, and species-specific dispersal ability and propensity [105,106]. Hence, large and mobile mammals tend to exhibit coarse-grained or widely clinal genetic structures, except when differentiation follows from behaviourally mediated dispersal limitation caused by, for example, individual-level habitat or resource specialization [87,107] or spatial clustering of related individuals [4,81]. Our analyses uncovered cryptic and remarkably fine-scaled genetic structure within the Saimaa ringed seal population, despite potentially high individual dispersal ability [19,21]. Small effective subpopulation sizes and the subdivided geography of the lake are undoubtedly central factors underlying this spatial structuring, but purely behavioural traits must also play a role, considering the differing levels of male- and female-mediated gene flow and the short distances that are involved. The fine-scaled differentiation within Lake Saimaa contrasts markedly with the population structures of marine Baltic and Arctic ringed seals, in which breeding colonies located hundreds or even thousands of kilometres apart often constitute essentially panmictic units [72,74]. Therefore, our results provide a striking demonstration that population structures of endangered animals cannot be predicted based on data from even closely related species or subspecies.

### Availability of supporting data

The microsatellite dataset supporting the results of this article is available in the Dryad repository ( http://datadryad.org), http://dx.doi.org/10.5061/dryad.5j754. The mtDNA sequence dataset is available from Genbank ( http://www.ncbi.nlm.nih.gov/genbank), accession numbers: JX109584–JX109833.

## Competing interests

The authors declare that they have no competing interests.

## Authors’ contributions

All authors contributed to designing the study. MK contributed to data collection, and MV conducted molecular analyses and compiled the dataset under the supervision of MR. MV, JUP, JA and TN analyzed the data. MV and TN wrote the manuscript, with significant editorial input from all co-authors. All authors read and approved the final manuscript.

## Supplementary Material

Additional file 1: Table S1 Microsatellite loci genotyped for Saimaa ringed seals. Click here for file

Additional file 2: Table S2 Microsatellite diversity indices for the spatial and temporal samples of the Saimaa ringed seal population. Click here for file

Additional file 3: Figure S1 Neighbourhood system created by TESS from collection-site coordinates of individual Saimaa ringed seals, after modification to improve its match to the topography of Lake Saimaa. Click here for file

Additional file 4: Figure S2 Population structure analysis of individual Saimaa ringed seals using Structure and TESS. (A) Mean log-likelihood for each number of *K* (number of clusters) from Structure runs. (B) Structure results post-processed with the Evanno approach; Δ*K* values for each *K*. (C) Deviance information criterion (DIC) scores computed by TESS plotted against *K.* Click here for file
