# Supplementary material for: Causes and consequences of fine-scale population structure in a critically endangered freshwater seal
Source: BMC Ecol. 2014 Jul 9;14:22. doi: 10.1186/1472-6785-14-22 (PMC4106222; doi:10.1186/1472-6785-14-22)
Supplement: Additional file 1: Table S1 — Microsatellite loci genotyped for Saimaa ringed seals. [file 1472-6785-14-22-S1.pdf]

Table S1 Microsatellite loci genotyped for Saimaa ringed seals.

Locus	Annealing t (°C)	PCR cycles	Concentration $MgCl_2$ (mM)	Label	Primer sequence	Repeat unit	GenBank accession nr	Reference
Hg1.4	55	30	1.5	6FAM	F: CTCCAAGACGACTGAAACCC<br>R: TACCATATCTTTGTGGCTCTG	(AC;GC)	AF055862	[23]
Hg2.3	55	30	1.5	NED	F: CCAATGACAACTTACTGAGAAT<br>R: TGTGAAGTGCTCTGTTTTGC	$(AC)_n$	AF055863	[24]
Hg3.6	58	30	1.5	PET	F: AGATCACATTCTTTTTATGGCTG<br>R: GATTGGATAAAGAAGATGTGAGGG	$(GT)_n$	G02089	[25]
Hg4.2	58	30	1.5	NED	F: AATCGAAATGCTGAGCCTCC<br>R: TGATTTGACTTCCCTTCCCTG	$(GT)_n$	G02090	[25]
Hg6.1	58	30	1.25	PET	F: TGCACCAGAGCCTAAGCAGACTG<br>R: CCACCAGCCAGTTCACCCAG	$(AC)_n$	G02091	[25]
Hg8.9	50	35	1.5	6FAM	F: TGTTAACTATCTGGCACAGAGTAAG<br>R: TTTCCTATGGGTTCTACTCTCAG	$(AC)_n$	G02094	[25]
Hg8.10	50	35	1.5	VIC	F: AATTCTGAAGCAGCCCAAG<br>R: GAATTCTTTTCTAGCATAGGTTG	$(AC)_n$	G02093	[25]
Hgdii	50	35	1.5	NED	F: ACCTGCCATAGTGCTCATC<br>R: AGGACTCCTGCCACTGAGAA	$(AC)_n$	G02095	[25, 26]
Hl15	50	35	1.5	NED	F: CATCTTGTAGTGCCAAAAAC<br>R: ATCTTTCAGTTGACCCTTCT	$(GT)_n$	AF140587	[27]
Pvc19	55	30	1.5	NED	F: GGGTGAACAGGATTTATCC<br>R: GTGCTAGATAACAATCCTAC	$(CA)_n$	L40989	[28]
Pvc26	53	35	1.5	NED	F: TTTTCTCCATACCTACATAAT<br>R: ATTGTGATCCCATTTTTGTAA	$(CA)_n$	L40988	[28]
Pvc30	50	35	1.5	6FAM	F: GCATGTGATCTTACAGCAAT<br>R: CATGGGTTCTCAATAGAAGA	$(TG)_nTA(TC)_nTA(TG)_n$	L40986	[28]
Pvc78	53	35	1.5	VIC	F: GAGTATACCTCCATACTACAC<br>R: AGTTGTTCTCCTGACCCAAG	$(AC)_n$	L40983	[28]
SGPv9	50	35	1.5	VIC	F: TAGTGTTTGGAAATGAGTTGGC<br>R: CTGATCCTTGTGAATCCCAGC	$(GT)_n$	G02096	[25]
SGPv10	55	30	1.5	VIC	F: TTCACTTAGCATAATTCCCTC<br>R: TCATGAATTGGTATTAGACAAAG	$(GT)_n$	U65443	[29]
SGPv11	55	30	1.5	6FAM	F: CAGAGTAAGCACCCAAGGAGCAG<br>R: GTGCTGGTGAATTAGCCCATTATAAG	$(AC)_n$	U65444	[29]
SGPv16	50	35	1.5	6FAM	F: AGCTAGTGTTAATGATGGTGTG<br>R: TCTGAGAGATTCAGAGTAACCTTC	$(AC)_n$	U65445	[29]
