# Supplementary material for: Causes and consequences of fine-scale population structure in a critically endangered freshwater seal
Source: BMC Ecol. 2014 Jul 9;14:22. doi: 10.1186/1472-6785-14-22 (PMC4106222; doi:10.1186/1472-6785-14-22)
Supplement: Additional file 2: Table S2 — Microsatellite diversity indices for the spatial and temporal samples of the Saimaa ringed seal population. [file 1472-6785-14-22-S2.pdf]

Table S2 Microsatellite diversity indices for the spatial and temporal samples of the Saimaa ringed seal population.

	Locus																	
Population	*Hg1.4*	*Hg2.3*	*Hg3.6*	*Hg4.2*	*Hg6.1*	*Hg8.9*	*Hg8.10*	*Hgdii*	*Hl15*	*Pvc19*	*Pvc26*	*Pvc30*	*Pvc78*	*SGPv9*	*SGPv10*	*SGPv11*	*SGPv16*	Mean
Lake Saimaa (N = 172)																		
N	172	172	172	172	172	172	172	172	171	172	171	170	172	172	172	172	172	
A	2	2	3	3	2	3	2	3	16	4	4	4	2	2	2	3	2	3.471
r	0.049	0.025	0.047	0.053	0.040	0.069	0.000	0.064	0.012	0.013	0.027	0.007	0.083	0.000	0.000	0.000	0.011	0.029
H_O	0.012	0.262	0.448	0.517	0.035	0.436	0.297	0.105	0.673	0.477	0.234	0.618	0.203	0.192	0.459	0.599	0.110	0.334
H_E	0.023	0.285	0.536	0.548	0.046	0.550	0.285	0.141	0.734	0.495	0.236	0.633	0.285	0.174	0.434	0.612	0.115	0.361
P	**0.018**	0.287	**0.006**	**0.000**	0.080	**0.008**	0.789	**0.007**	**0.006**	0.180	**0.033**	0.240	**0.001**	0.370	0.483	0.637	0.477	**< 0.001**
Northern Saimaa (N = 15)																		
N	15	15	15	15	15	15	15	15	15	15	15	14	15	15	15	15	15	
A	1	2	2	3	1	2	2	2	6	3	2	3	2	2	2	3	1	2.294
r	0.001	0.000	0.000	0.050	0.001	0.017	0.111	0.000	0.000	0.092	0.000	0.000	0.155	0.000	0.000	0.000	0.001	0.025
H_O	0	0.400	0.533	0.467	0	0.467	0.133	0.067	0.733	0.200	0.333	0.643	0.267	0.067	0.667	0.733	0	0.336
H_E	-	0.331	0.515	0.508	-	0.508	0.239	0.067	0.720	0.297	0.287	0.521	0.515	0.067	0.515	0.600	-	0.335
P	-	1.000	1.000	0.059	-	1.000	0.203	1.000	0.872	0.324	1.000	0.727	0.115	1.000	0.327	0.311	-	0.639
Haukivesi area (N = 99)																		
N	99	99	99	99	99	99	99	99	98	99	99	99	99	99	99	99	99	
A	1	2	3	3	1	3	2	3	15	3	4	4	2	2	2	3	2	3.235
r	0.001	0.012	0.111	0.063	0.001	0.086	0.005	0.067	0.000	0.025	0.000	0.000	0.052	0.000	0.003	0.000	0.011	0.026
H_O	0	0.212	0.354	0.495	0	0.414	0.232	0.172	0.827	0.424	0.333	0.657	0.253	0.323	0.414	0.657	0.152	0.348
H_E	-	0.222	0.536	0.557	-	0.541	0.237	0.225	0.833	0.468	0.309	0.660	0.305	0.272	0.421	0.645	0.158	0.376
P	-	0.647	**0.000**	**0.002**	-	**0.002**	1.000	0.051	0.097	0.208	0.819	**0.021**	0.099	0.069	1.000	0.758	0.530	**0.000**
Pihlajavesi area (N = 43)																		
N	43	43	43	43	43	43	43	43	43	43	42	42	43	43	43	43	43	
A	2	2	3	2	2	3	2	1	7	4	2	4	2	1	2	3	2	2.588
r	0.085	0.027	0.000	0.000	0.000	0.051	0.000	0.001	0.021	0.000	0.105	0.000	0.000	0.001	0.000	0.000	0.000	0.017
H_O	0.047	0.395	0.558	0.605	0.023	0.465	0.465	0	0.302	0.674	0.000	0.548	0.116	0	0.535	0.465	0.047	0.309
H_E	0.090	0.436	0.567	0.499	0.023	0.573	0.361	-	0.332	0.581	0.047	0.557	0.111	-	0.436	0.418	0.046	0.299
P	0.070	0.722	0.884	0.217	1.000	0.164	0.083	-	0.204	0.391	**0.012**	0.841	1.000	-	0.167	0.915	1.000	0.144

	Locus																	
Population	*Hg1.4*	*Hg2.3*	*Hg3.6*	*Hg4.2*	*Hg6.1*	*Hg8.9*	*Hg8.10*	*Hgdii*	*Hl15*	*Pvc19*	*Pvc26*	*Pvc30*	*Pvc78*	*SGPv9*	*SGPv10*	*SGPv11*	*SGPv16*	Mean
Southern Saimaa (*N* = 15)																		
N	15	15	15	15	15	15	15	15	15	15	15	15	15	15	15	15	15	
A	1	2	3	2	2	3	2	1	7	3	2	4	2	1	2	3	2	2.471
r	0.001	0.000	0.000	0.000	0.022	0.000	0.000	0.001	0.000	0.000	0.000	0.000	0.000	0.001	0.022	0.000	0.000	0.003
H_O	0	0	0.667	0.467	0.333	0.467	0.400	0	0.667	0.533	0.133	0.533	0	0	0.333	0.467	0.133	0.310
H_E	-	0.067	0.480	0.370	0.370	0.480	0.405	-	0.697	0.522	0.129	0.522	0.067	-	0.370	0.439	0.129	0.297
P	-	1.000	0.309	0.528	1.000	0.438	1.000	-	0.780	1.000	1.000	0.507	1.000	-	1.000	0.718	1.000	0.999
1980s (*N* = 59)																		
N	59	59	59	59	59	59	59	59	58	59	59	59	59	59	59	59	59	
A	2	2	3	3	2	3	2	3	13	4	4	4	2	2	2	3	2	3.294
r	0.089	0.005	0.048	0.045	0.000	0.044	0.000	0.084	0.004	0.001	0.000	0.000	0.086	0.000	0.000	0.000	0.042	0.026
H_O	0.000	0.288	0.424	0.542	0.017	0.458	0.271	0.102	0.741	0.508	0.271	0.746	0.254	0.186	0.475	0.542	0.119	0.350
H_E	0.034	0.295	0.509	0.578	0.017	0.548	0.261	0.158	0.789	0.523	0.266	0.648	0.356	0.171	0.428	0.559	0.142	0.369
P	0.009	1.000	0.314	0.091	1.000	0.345	1.000	**0.043**	0.117	0.450	1.000	0.419	0.056	1.000	0.540	0.789	0.281	0.082
1990s (*N* = 48)																		
N	48	48	48	48	48	48	48	48	48	48	48	48	48	48	48	48	48	
A	2	2	3	3	2	3	2	3	14	4	3	4	2	2	2	3	2	3.294
r	0.000	0.000	0.084	0.046	0.082	0.077	0.000	0.078	0.000	0.002	0.075	0.008	0.049	0.000	0.000	0.000	0.000	0.029
H_O	0.021	0.313	0.396	0.563	0.042	0.375	0.313	0.146	0.708	0.417	0.208	0.667	0.208	0.208	0.479	0.625	0.083	0.339
H_E	0.021	0.295	0.547	0.568	0.081	0.508	0.266	0.207	0.755	0.438	0.257	0.681	0.252	0.189	0.468	0.634	0.081	0.367
P	1.000	1.000	0.100	**0.004**	0.063	0.135	0.577	0.095	0.156	0.710	**0.010**	0.381	0.242	1.000	1.000	0.128	1.000	**0.010**
2000s (*N* = 65)																		
N	65	65	65	65	65	65	65	65	65	65	64	63	65	65	65	65	65	
A	2	2	3	3	2	3	2	3	12	3	2	4	2	2	2	3	2	3.059
r	0.000	0.076	0.017	0.056	0.000	0.072	0.013	0.000	0.010	0.020	0.000	0.072	0.097	0.000	0.000	0.000	0.000	0.025
H_O	0.015	0.200	0.508	0.462	0.046	0.462	0.308	0.077	0.585	0.492	0.219	0.460	0.154	0.185	0.431	0.631	0.123	0.315
H_E	0.015	0.273	0.553	0.502	0.045	0.578	0.322	0.075	0.664	0.510	0.196	0.580	0.240	0.169	0.417	0.635	0.116	0.347
P	1.000	**0.049**	0.100	**0.014**	1.000	0.069	0.705	1.000	**0.022**	0.310	1.000	0.139	**0.013**	1.000	1.000	0.986	1.000	0.066

A = number of alleles, *r* = null allele frequency, H_O = observed heterozygosity, H_E = expected heterozygosity

P-values (uncorrected) in **bold** indicate significant deviations from Hardy-Weinberg equilibrium
