# Supplementary material for: Causes and consequences of fine-scale population structure in a critically endangered freshwater seal
Source: BMC Ecol. 2014 Jul 9;14:22. doi: 10.1186/1472-6785-14-22 (PMC4106222; doi:10.1186/1472-6785-14-22)
Supplement: Additional file 3: Figure S1 — Neighbourhood system created by TESS from collection-site coordinates of individual Saimaa ringed seals, after modification to improve its match to the topography of Lake Saimaa. [file 1472-6785-14-22-S3.pdf]

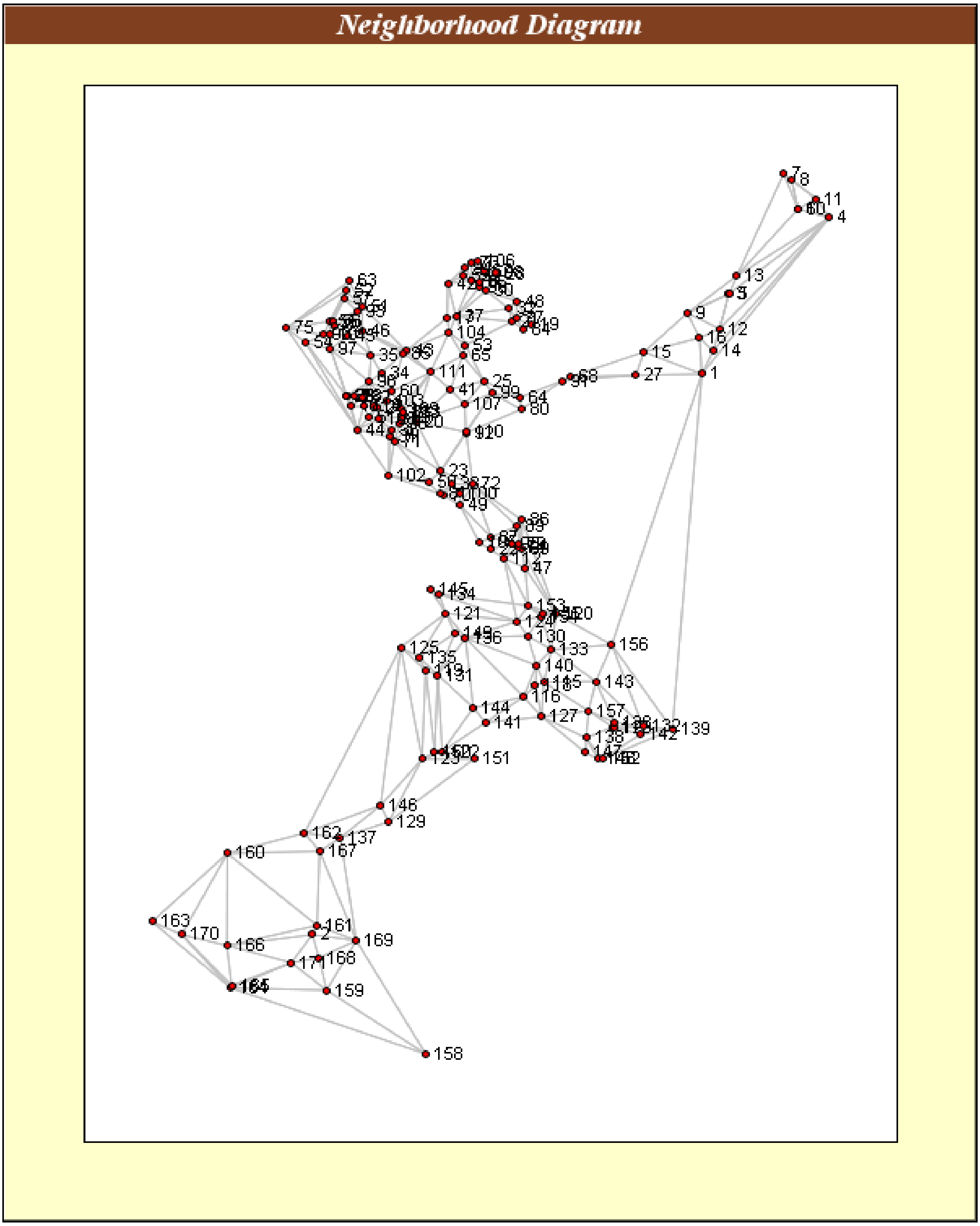

Figure S1 Neighbourhood system created by TESS from collection-site coordinates of individual Saimaa ringed seals, after modification to improve its match to the geography of Lake Saimaa.
