# Supplementary material for: Causes and consequences of fine-scale population structure in a critically endangered freshwater seal
Source: BMC Ecol. 2014 Jul 9;14:22. doi: 10.1186/1472-6785-14-22 (PMC4106222; doi:10.1186/1472-6785-14-22)
Supplement: Additional file 4: Figure S2 — Population structure analysis of individual Saimaa ringed seals using Structure and TESS. (A) Mean log-likelihood for each number of K (number of clusters) from Structure runs. (B) Structure results post-processed with the Evanno approach; ΔK values for each K. (C) Deviance information criterion (DIC) scores computed by TESS plotted against K. [file 1472-6785-14-22-S4.pdf]

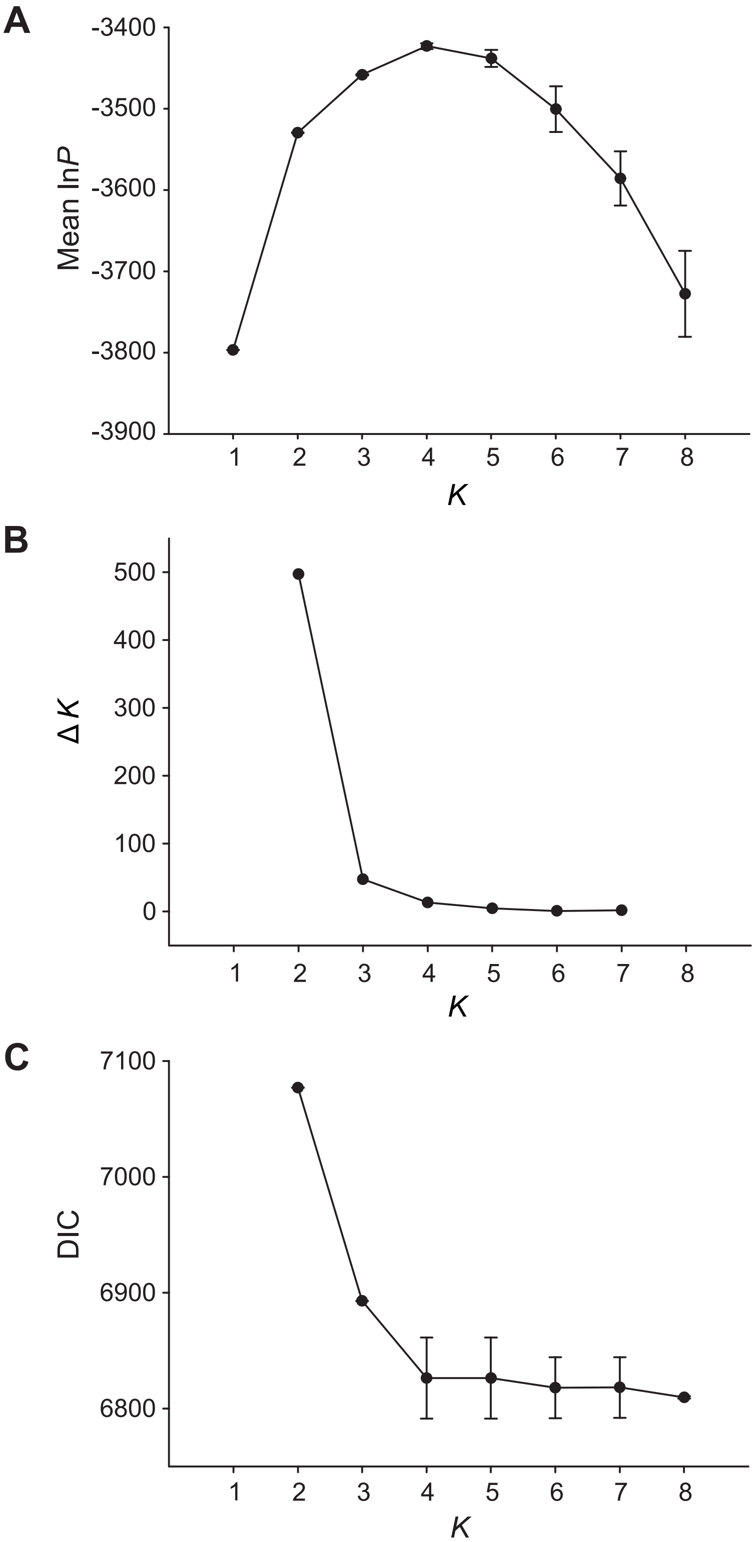

Figure S2 Population structure analysis of individual Saimaa ringed seals using Structure and TESS. (**A**) Mean log-likelihood for each number of *K* (number of clusters) from Structure runs. (**B**) Structure results post-processed with the Evanno approach; Δ*K* values for each *K*. (**C**) Deviance information criterion (DIC) scores computed by TESS plotted against K.
